# Antibody-secreting cell destiny emerges during the initial stages of B-cell activation

**DOI:** 10.1038/s41467-020-17798-x

**Published:** 2020-08-10

**Authors:** Christopher D. Scharer, Dillon G. Patterson, Tian Mi, Madeline J. Price, Sakeenah L. Hicks, Jeremy M. Boss

**Affiliations:** Department of Microbiology and Immunology and the Emory Vaccine Center, Emory University School of Medicine, Atlanta, GA 30322 USA

**Keywords:** Humoral immunity, Epigenetics in immune cells, Plasma cells, Transcriptomics

## Abstract

Upon stimulation, B cells assume heterogeneous cell fates, with only a fraction differentiating into antibody-secreting cells (ASC). Here we investigate B cell fate programming and heterogeneity during ASC differentiation using T cell-independent models. We find that maximal ASC induction requires at least eight cell divisions in vivo, with BLIMP-1 being required for differentiation at division eight. Single cell RNA-sequencing of activated B cells and construction of differentiation trajectories reveal an early cell fate bifurcation. The ASC-destined branch requires induction of IRF4, MYC-target genes, and oxidative phosphorylation, with the loss of CD62L expression serving as a potential early marker of ASC fate commitment. Meanwhile, the non-ASC branch expresses an inflammatory signature, and maintains B cell fate programming. Finally, ASC can be further subseted based on their differential responses to ER-stress, indicating multiple development branch points. Our data thus define the cell division kinetics of B cell differentiation in vivo, and identify the molecular trajectories of B cell fate and ASC formation.

## Abstract (web summary)

The development of activated B cells into antibody-secreting cells (ASC) is a critical step for humoral immunity. Here the authors show, using adoptive transfers and single cell RNA sequencing, that commitment to ASC occurs soon following B cell activation, and is coordinated by specific transcriptome programs and proliferation kinetics.

## Introduction

Many pathogens are subject to immunological control via the generation of antibody-secreting cells (ASC) or plasma cells. After being activated by external stimuli, resting naive B (nB) cells rapidly proliferate, and it is now appreciated that cell division is an essential component of ASC differentiation^1,2^. A core feature of the humoral immune response is the predictable kinetics of B-cell expansion and differentiation^3^. Ex vivo, a stochastic model of differentiation predicts population level immune responses, while accounting for differentiation heterogeneity between individual cells^4–7^. Part of the mechanism is dependent on the expression levels of MYC, which is influenced by immune stimulation, where MYC functions as a division-independent temporal timer controlling the number of cell divisions^8–10^. Importantly, examination of the cell fate outcomes of individual cells indicated that sibling cells have similar fates compared to unrelated cells that have undergone the same number of divisions^5,6^. Thus, considerable heterogeneity exists between responding B cells but the differences in transcriptional programming that drives this heterogeneity is unknown.

In addition to cell division, ASC differentiation requires the coordinated regulation of hundreds of genes, ultimately demanding differentiating cells to extinguish the B-cell program and initiate the ASC program^11,12^. In vivo, transcriptional rewiring and epigenetic remodeling^13^ occur within the framework of cell division^14–18^. For example, DNA hypomethylation events occur in ASC predominately at enhancers and this is accompanied by a change in chromatin accessibility and gene expression^14,16^. Reciprocally, de novo DNA methylation is critical for limiting B-cell activation and plasma cell differentiation^19^. Epigenetic chromatin modifiers, including EZH2 and LSD1, alter the division and differentiation kinetics of B cells in vivo^15,20^. While these studies underscore the importance of epigenetic reprogramming during B-cell differentiation and how these reprogramming events help coordinate the appropriate ASC transcriptional program, they have not examined how cellular division and differentiation occur at the single cell (sc) level.

Using scRNA-seq, we explore the molecular reprogramming that lead to heterogenous cell fate outcomes during B-cell differentiation to T cell-independent antigens in vivo. We find that B-cell differentiation in vivo requires a minimum of eight cell divisions in the absence of cell extrinsic signals. In addition, we define a path to ASC that reveal an early decision point, with one branch leading to ASC differentiation that is dependent on IRF4. Cells that follow this branch induce gene sets that included oxidative phosphorylation (OXPHOS), cell proliferation based on MYC, and ultimately endoplasmic reticulum (ER)-stress responses. Cells that follow the second branch induced inflammatory gene sets and fail to downregulate L-selectin (CD62L) and form ASCs. Thus, we provide a molecular path that defines the variation in cell fate decision making that is required to form an ASC.

## Results

### Cell division is critical for in vivo B-cell differentiation

**Fig. 1:**
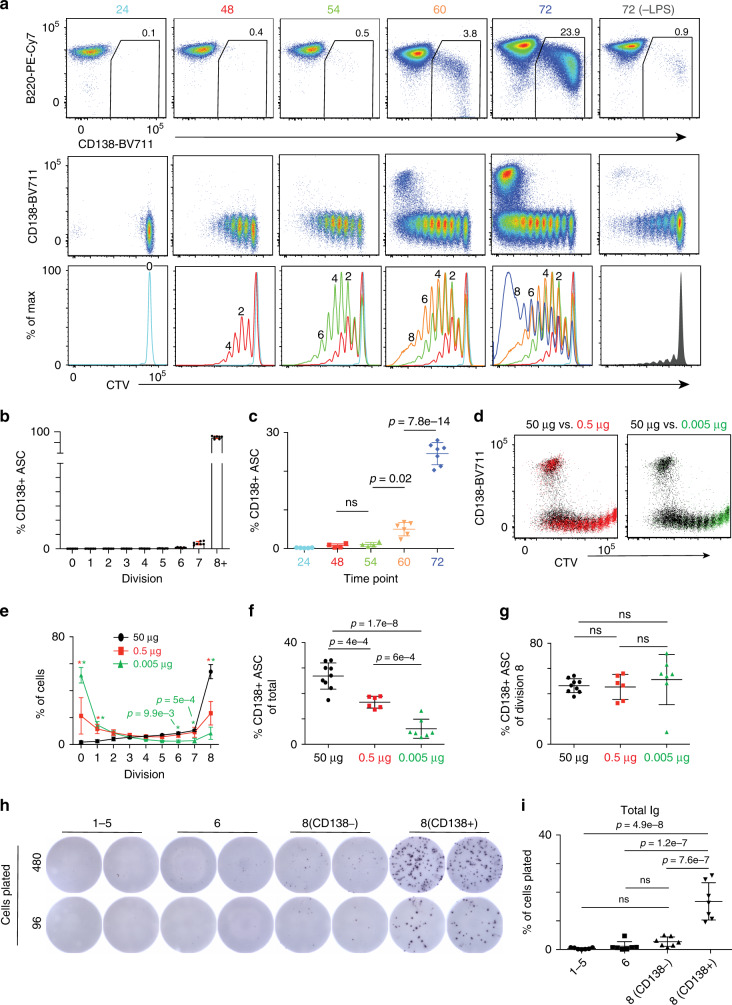
Cell division mapping of ASC differentiation in vivo. **a** Representative flow cytometry plots of B220 versus CD138 (top), CD138 versus CTV (middle), and CTV histograms (bottom) at the indicated time points following in vivo LPS inoculation. The percentage of total CD138^+^ cells is indicated in the top row. **b** Frequency of CD138^+^ cells at each division at 72 h. **c** Frequency of CD138^+^ cells at each time point. **d** Representative flow cytometry of CD138 versus CTV plots 72 h after μMT hosts were challenged with 50 μg (black), 0.5 μg (red), or 0.005 μg (green) of LPS. **e** Frequency of transferred cells at each division for each dose. Red or green asterisk indicates significance of *P* < 0.0001 between 50 versus 0.5 μg and 50 versus 0.005 μg, respectively, unless otherwise stated. **f** The percentage of total CD138^+^ cells is shown for samples from (**e**). **g** The percentage of division 8 cells that are CD138^+^ is shown for samples from (**e**). **h** Representative images of total Ig ELISPOTs on cells sorted from division 1–5, 6, or 8 (CD138^–^ or CD138^+^) 72 h post LPS challenge from one mouse. **i** ELISPOTs from (**h**) were quantitated as the percentage of total cells plated that formed ASC. Data in (**a**–**c**) are representative of three independent experiments containing 24 h (*n* = 5), 48 h (*n* = 4), 54 h (*n* = 4), 60 h (*n* = 6), and 72 h (*n* = 7) mice. Data in (**d**–**g**) were derived from three independent experiments with 50 μg (*n* = 9), 0.5 μg (*n* = 6), and 0.005 μg (*n* = 7) mice. Data in (**h**–**i**) were derived from two independent experiments from seven total mice. Data in (**b**, **c**, **e**–**g**, **i**) represent mean ± SD. Statistical significance in (**e**) was determined by a two-way ANOVA with Tukey’s post test for multiple comparisons. Statistical significance for (**c**, **f**, **g**, **i**) was determined by one-way ANOVA with Tukey’s post test for multiple comparisons. Source data are provided as a Source Data file.

The modeling of B-cell differentiation kinetics in the context of cell division has largely been performed using ex vivo stimulated cells^4–6,8,9,21–23^. To better understand the kinetics of cell division with the timing of B-cell differentiation in vivo, we used an adoptive transfer model. Here, CellTrace Violet (CTV)-labeled splenic CD43^–^ B cells from CD45.2^+^ donor mice were transferred to CD45.1^+^ μMT mice. After 1 day, host μMT were inoculated with 50 μg of LPS and cell division/differentiation was assessed by the expression of the surface proteoglycan molecule Syndecan-1 (CD138)^24,25^ in a detailed time course over the next 3 days^16^. At 24 h, no division was observed, defining a minimum lag time before responding cells begin dividing (Fig. 1a). Between 48 and 54 h, a maximum of seven cell divisions were observed with no significant increase in the percentage of CD138^+^ cells. However, by 60 h, a small fraction of the cells divided at least eight times and the first CD138^+^ cells were observed (Fig. 1a). At 72 h, more cells reached or exceeded the eight division, corresponding to a peak in CD138^+^ cells or ASC (Fig. 1b). As such, ASC differentiation corresponds with the time it takes for responding B cells to reach division 8 (Fig. 1c). Mice that received CTV-labeled cells and no LPS displayed background, homeostatic levels of proliferation and differentiation at 72 h (Fig. 1a).

To determine whether LPS dosage influenced in vivo ASC differentiation, a dose comparison covering a 10,000-fold range was performed. At all doses, a minimum of eight divisions were still required for ASC formation (Fig. 1d). Similar to ex vivo differentiation^8^, lower dosages impacted the magnitude of the overall response, ultimately resulting in fewer cells reaching division 8 and forming ASC (Fig. 1e, f). However, for cells reaching division 8, the same proportion differentiates to ASC (Fig. 1g). Thus, these data indicate that in vivo LPS dosage affects the number of responding B cells, but not the requirement of eight divisions for ASC formation. Finally, to confirm that only the CD138^+^ cells at division 8 were indeed ASC, we performed ELISPOT assays on cells sorted from divisions 1–5, 6, and 8. Division 8 cells were split based on CD138 expression. Only cells that had reached division 8 and were CD138^+^ secreted robust levels of antibodies (Fig. 1h, i). Although not all CD138^+^ cells formed spots, this was not unexpected as a loss in secretion of some cells has been observed in ELISPOT assays following cell sorting^26^.

### B cell division kinetics for T-independent stimuli

**Fig. 2:**
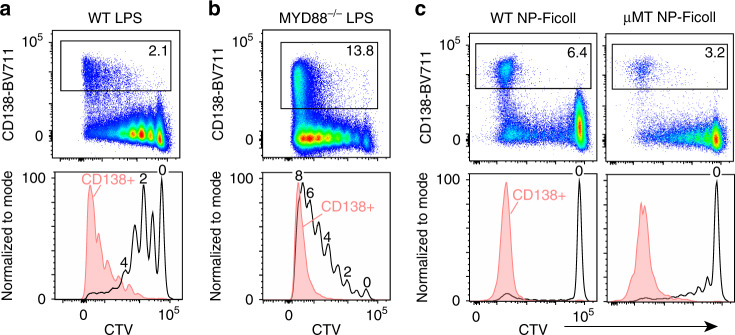
T-independent type I and II antigens reveal similar division requirements. Representative flow cytometry of CD138 versus CTV plots (top) and CTV histograms (bottom) 72 h post immune challenge for **a** WT hosts challenged with 50 μg of LPS; **b** MYD88^–/–^ hosts challenged with 50 μg of LPS; **c** WT hosts (left) or μMT hosts (right) challenged with 50 μg of NP-Ficoll. Black CTV histograms represent distribution of total transferred cells, while solid red CTV histograms represent CD138^+^ cells. Gating and percentage of CD138^+^ cells are indicated in the top row. Data in (**a**, **c**) for WT hosts are representative examples from three independent experiments and six total mice each. Data in (**b**) are representative of two independent experiments with six total mice. Data in (**c**) for μMT host are representative of two independent experiments with six total mice.

To determine whether similar division and differentiation kinetics were observed in a wild-type (WT) host setting, CTV-labeled B cells from CD45.1^+^ donor mice were transferred into CD45.2^+^ C57BL/6J WT hosts. After 1 day, hosts were inoculated with 50 μg LPS and division/differentiation was assessed on day 3. In WT hosts, we found the cell division kinetics of ASC differentiation to be altered such that CD138^+^ cells were predominantly observed in division 8 but were also observed as early as division 3 (Fig. 2a). To determine if the observed differences in differentiation kinetics were due to cell extrinsic effects from host cells also responding to LPS, the adoptive transfer was repeated using *Myd88*-deficient hosts (MYD88^–/–^) that cannot respond to LPS^27^. In MYD88^–/–^ hosts, cell division and differentiation kinetics closely resembled what we observed for μMT hosts, with CD138^+^ cells in division 8, as well as a similar distribution of cells across all divisions (Fig. 2b). This suggests that cell extrinsic effects from LPS-responding host cells can impact the division in which differentiation occurs.

To determine whether similar division kinetics were observed for other stimuli, adoptive transfers using WT and μMT hosts were performed followed by challenge with the T cell-independent type II antigen 4-hydroxy-3-nitrophenylacetyl (NP)-Ficoll. Here, a similar division/differentiation requirement in these mice as seen in μMT and MYD88^–/–^ hosts challenged with LPS was observed (Fig. 2c). Collectively, these results indicate that there is a similar cell division requirement during B-cell differentiation in response to T cell-independent type I and II antigens.

### BLIMP-1-dependent reprogramming is defective at division 8

**Fig. 3:**
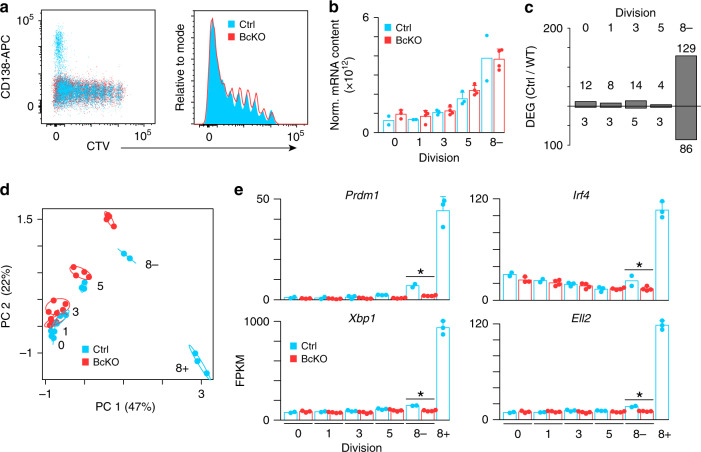
BLIMP-1-dependent transcriptional reprogramming is defective in division 8. (**a**) Representative flow cytometry analysis of *Prdm1*^fl/fl^ (Ctrl) and *Cd19*^Cre/+^*Prdm1*^fl/fl^ (BcKO) B cells 72 h after LPS inoculation. (**b**) ERCC normalized total mRNA content for samples from the indicated divisions sorted from (**a**). **c** Bar plot showing the number of differentially expressed genes (DEG) for the indicated division between Ctrl and BcKO B cells. **d** Principal component analysis of samples from (**b**) using all DEG. The location of cells in each division is labeled. Circles denote 99% confidence intervals for samples in each division. **e** Bar plot of expression of select genes from divisions 0, 1, 3, 5, 8^–^ and for Ctrl 8^+^. FPKM fragments per kilobase per million. Data in (**b**, **e**) represent mean ± SD and asterisk indicates statistical significance (FDR < 0.05) as determined by edgeR^59^. Data were derived from 2 to 4 individual mice as follows: Ctrl division 0 (*n* = 2), 1 (*n* = 2), 3 (*n* = 3), 5 (*n* = 3), 8− (*n* = 2), and 8+ (*n* = 3); BcKO division 0 (*n* = 3), 1 (*n* = 4), 3 (*n* = 4), 5 (*n* = 4), 8− (*n* = 4). Source data are provided as a Source Data file.

The transcription factor BLIMP-1, encoded by *Prdm1*, is essential for ASC differentiation^28^ but is dispensable for the early proliferative phase of activated B cells (actB)^17^. Similar to previous results^17^, adoptive transfers using CTV-labeled *Cd19*^Cre/+^*Prdm1*^fl/fl^ (BcKO) or *Prdm1*^fl/fl^ (Ctrl) splenic B cells followed by stimulation with LPS as above revealed that BcKO B cells divided the same number of times and were distributed similarly across the divisions compared to Ctrl B cells (Fig. 3a). However, no CD138^+^ ASC were observed in the BcKO adoptive transfer, despite cells reaching the eight division. To explore the timing and extent of BLIMP-1-dependent reprogramming with respect to division number, we FACS isolated and performed RNA-seq on Ctrl and BcKO responding B cells from divisions 0, 1, 3, 5, as well as cells in the eight division that were CD138^–^ (8−) and from Ctrl mice, CD138^+^ division 8 (8+) cells. Similar increases in total mRNA content were observed irrespective of BLIMP-1 status (Fig. 3b). *Prdm1* expression was first detected in 8− and peaked in 8+ B cells following differentiation^16^. Consistent with this expression pattern, the majority of differentially expressed genes (DEG) between Ctrl and BcKO were observed in 8− B cells (Fig. 3c). Principal component analysis (PCA) of all DEG separated samples primarily by division status with the largest variation between Ctrl and BcKO occurring in 8− cells. (Fig. 3d). Examples of genes that failed to be induced in BcKO 8− cells included those that are known to be critical for ASC differentiation and regulated by BLIMP-1 such as *Xbp1*, *Irf4*, and *Ell2*^29,30^ (Fig. 3e). Thus, BLIMP-1-dependent reprogramming is initiated no earlier than division 5 with molecular defects observed in division 8 cells.

### ScRNA-seq captures a continuum of LPS-responding B cells

**Fig. 4:**
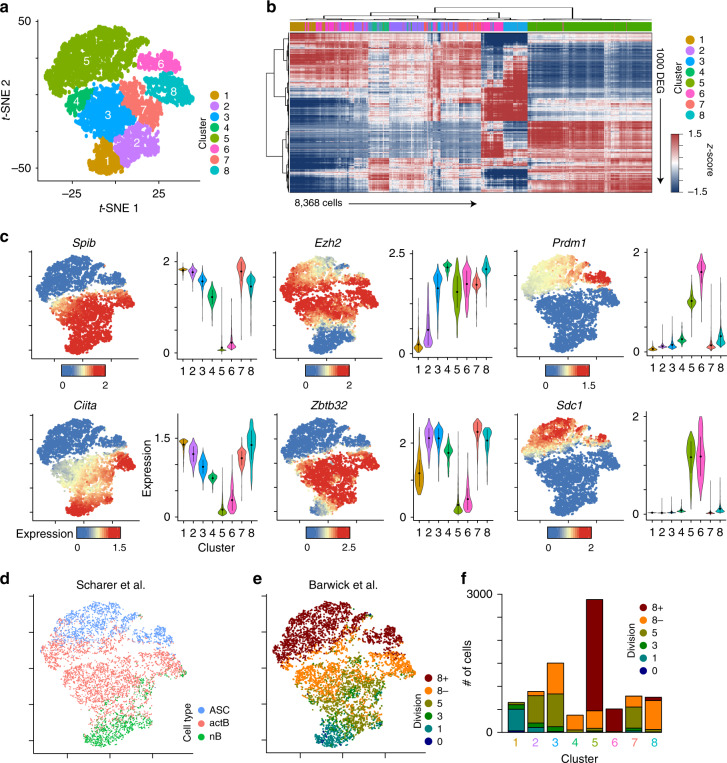
scRNA-seq reveals a continuum of heterogeneous LPS-responding B cells. **a***t*-SNE plot of 8368 WT cells responding to LPS showing eight distinct clusters of cells. **b** Heatmap showing hierarchical clustering of the top 1000 differentially expressed genes (DEG) between cells in each of the eight clusters from (**a**). Data represent *z*-score normalized MAGIC expression values. **c***t*-SNE plot (left) from (**a**) and violin plots (right) for each cluster showing the MAGIC gene expression data for the indicated gene. For violin plots, the dots represent the mean and lines represent first and third quartile ranges. **d***t*-SNE plot from (**a**) showing the annotation of each cell with naïve B cells (nB) and ex vivo differentiated activated B cells (actB) and ASC from Scharer et al.^14^. **e***t*-SNE plot from (**a**) showing the annotation of each cell with division sorted LPS-responding B cells from Barwick et al.^16^. **f** Bar plot quantitating the number of cells from each cluster annotated to specific divisions in (**e**). Data represent combined cells from two independent mice. Source data are provided as a Source Data file.

Molecular analysis of discrete divisions highlights the progressive epigenetic and transcriptional reprogramming that occur as B cells divide^14,16^. However, B cells respond asynchronously and only a fraction of B cells that make it to division 8 differentiate, indicating there may be additional mechanisms that instruct B-cell fate decisions. To resolve B-cell differentiation at a finer molecular resolution, we performed scRNA-seq on adoptively transferred cells in μMT hosts at 72 h post LPS challenge (Supplementary Fig. 1). In total, mRNA from 8368 B cells was sequenced from two independent mice. In agreement with the previous division data^16^, overall mRNA content increased among responding B cells (Supplementary Fig. 2). LPS-responding B cells were grouped into eight distinct clusters and visualized by *t*-distributed stochastic neighbor-embedded (*t*-SNE) projections (Fig. 4a). Hierarchical clustering of cells using the top 1000 DEG between all clusters indicated that cells in cluster 5 were the most transcriptionally distinct (Fig. 4b). Strikingly, we observed variation between individual cells within each cluster, revealing a heterogeneity not previously appreciated.

To assign clusters to cell types, we assessed the expression of both single genes that define B cells, actB cells and ASC^12^, as well as global transcriptome signatures of each purified population^14^. B-cell transcription factors, such as *Spib*, *Ciita*, and *Pax5*, were expressed in clusters 1–4 and 7–8 (Fig. 4c and Supplementary Fig. 2). ActB genes, such as *Zbtb32*, *Aicda*, and *Ezh2*, were expressed in clusters 2–4, 7, and 8. ASC genes, such as *Sdc1* (CD138), *Jchain*, and *Irf4*, were highly expressed in clusters 5 and 6. Using previously published RNA-seq datasets containing transcriptome profiles of CD43^–^ nB cells and ex vivo LPS-responding actB and ASC^14^, we assigned each cell to one of these three differentiation stages using a K-nearest neighbor (KNN) approach. Cluster 1 cells were classified as nB and those in clusters 2–4, 7, and 8 as actB (Fig. 4d). Cluster 5 and 6 cells were primarily classified as ASC with a subset of cluster 5 bearing hallmarks of actB. Importantly, the number of cells assigned to these clusters closely matched the frequency of CD138^+^ ASC observed by flow cytometry (Supplementary Fig. 2).

To further annotate the clusters with respect to cell division, we compared the transcriptional state of each cell to gene expression data for LPS-responding B cells at divisions 0, 1, 3, 5, and 8− and 8+^16^ using KNN. This analysis reaffirmed that cells assigned to cluster 1 represented those that were in the earliest stages of differentiation and cells assigned to clusters 5 and 6 represented B cells that have differentiated (Fig. 4e, f). Furthermore, we found that the transcriptome of cells assigned to actB aligned with gene expression data of LPS-responding B cells in divisions 3, 5, and 8−. These analyses indicate a continuum/progression of cellular differentiation from nB (cluster 1) through dividing actB (clusters 2–4, 7, and 8) to ASC (clusters 5 and 6), capturing the initial, intermediate, and end stages of B-cell differentiation in response to LPS.

### Pseudotime analysis defines divergent differentiation paths

**Fig. 5:**
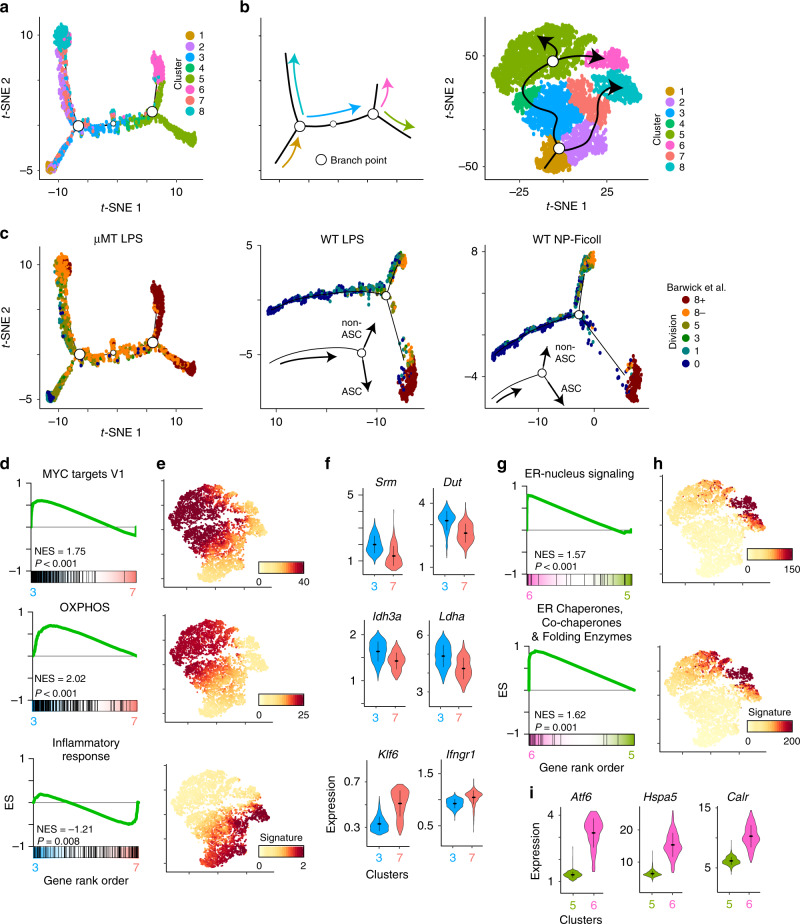
Pseudotime identifies divergent activated B-cell differentiation trajectories. **a***t*-SNE plot of pseudotime ordered cells from Fig. 4a showing the location of cells from each cluster. Open circles denote pseudotime branch points. **b** Schematic showing the pseudotime order of cells from (**a**) (left) and from the *t*-SNE plot from Fig. 4a (right). **c** Pseudotime *t*-SNE plot from (**a**) (left) or from scRNA-seq data on adoptively transferred cells responding to LPS (middle) or NP-Ficoll (right) in WT hosts showing cells annotated based on division sorted LPS-responding B cells from Barwick et al.^16^ (see Fig. 4e and Supplementary Fig. 1). Circles denote pseudotime branch points. **d** GSEA of the indicated datasets comparing the transcriptional profile between cells in clusters 3 and 7. **e***t*-SNE plot showing mean MAGIC expression levels for all Leading Edge genes from the GSEA gene set in (**d**). **f** Violin plots showing MAGIC expression levels for cells in clusters 3 and 7 for the indicated genes. Genes were chosen from the adjacent GSEA gene set from (**d**). Within each violin plot, the dots represent mean MAGIC expression levels and lines represent first and third quartile ranges. **g** GSEA of the indicated datasets comparing the transcriptional profile of cells in clusters 5 and 6. **h***t*-SNE plot showing mean MAGIC expression levels for all Leading Edge genes from the adjacent GSEA gene set in (**g**). The ER chaperones, co-chaperones, and folding enzymes gene set were described previously^34^. **i** Violin plots of select genes representative of gene sets in (**g**). WT LPS and WT NP-Ficoll scRNA-seq data in (**c**) represent combined cells from two independent mice. Significance for GSEA was calculated by permutation testing with *P* < 0.001 indicating the gene set was enriched over all 1000 permutations. Source data are provided as a Source Data file.

To construct potential differentiation trajectories and understand the progression between cellular states, we computationally ordered cells along pseudotime^31^. Strikingly, this analysis revealed two distinct branch points as cells proceeded along pseudotime and differentiated from nB to ASC (Fig. 5a, b). Analyzing the branching trajectory with cell division assignments revealed that the first branch point bifurcated actB into two groups, with one branch proceeding to CD138^+^ ASC and the other remaining as B cells (Fig. 5b). We have termed these the ASC-destined and non-ASC branches, respectively. The ASC-destined branch includes clusters 5 and 6 and cells that are in division 8+ and possess all the hallmark features of ASC (Fig. 5b). The non-ASC branch terminates in clusters 7 and 8 and includes cells that have divided at least five to eight times but have not differentiated further. To corroborate these observations using different host and stimuli, B cells were adoptively transferred to WT hosts and stimulated with both LPS and NP-Ficoll. At 72 h, all responding cells were isolated and scRNA-seq was performed as above (Supplementary Fig. 1). For each dataset, a pseudotime trajectory was constructed and each cell assigned both a cell-division and cell-type phenotype as with the μMT host data. This analysis revealed the presence of a branch point that separated actB into an ASC-destined and non-ASC trajectory (Fig. 5c and Supplementary Fig. 2).

To identify the properties of the different branches, gene set enrichment analysis (GSEA)^32^ was performed on cell clusters just after the bifurcation point (clusters 3 and 7). This indicated that cells in the ASC-destined branch upregulated genes associated with MYC activation and OXPHOS (Fig. 5d–f), both of which are important for achieving the metabolic and catabolic requirements during plasma cell differentiation^17,33^. Examples within the ASC-destined trajectory include expression of *Srm*, *Dut*, *Idh3a*, and *Ldha*. Conversely, cells along the non-ASC branch displayed higher expression of genes, such as *Klf6* and *Ifngr1*, which were associated with an inflammatory response and were upregulated in cluster 7 and maintained in cluster 8.

A second branch point following CD138^+^ ASC differentiation was identified in the μMT dataset within clusters 5 and 6 (Fig. 5a–c). GSEA between these clusters indicated that cells in cluster 6 were enriched for genes involved in ER-to-nucleus signaling and ER chaperones, co-chaperones and folding enzymes^34^ (Fig. 5g, h). Examples of genes upregulated in cluster 6 included *Atf6*, *Hspa5*, and *Calr* (Fig. 5i). Although both clusters 5 and 6 have upregulated the ER stress response factor *Xbp1*, these data suggest that ASC in cluster 6 were under distinct ER stress.

### Multiple factors regulate the ASC-destined branch

**Fig. 6:**
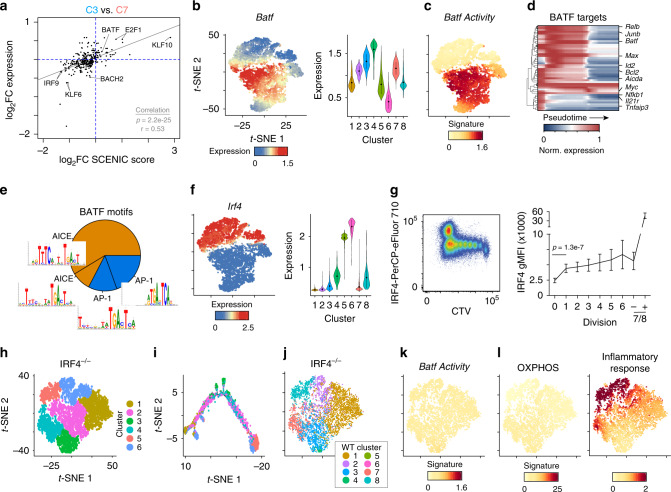
IRF4 is critical for establishing the ASC-destined branch transcriptional program. **a** Scatter plot showing the expression log_2_ fold change (log_2_FC) versus the log_2_FC in SCENIC activity score between cluster 3 versus 7 for each transcription factor. Gray line represents linear regression with significance determined by one-way ANOVA. Pearson’s *r* correlation is indicated. **b***t*-SNE plot (left) from Fig. 4a and violin plot (right) showing the MAGIC gene expression data for the indicated gene. For violin plots, dots represent mean and lines represent first and third quartile ranges. **c***t*-SNE plot showing mean MAGIC expression levels for BATF target genes determined by SCENIC. **d** Heatmap showing expression of 46 BATF target genes along pseudotime in WT cells. For each gene, the mean MAGIC expression level is normalized to the maximal value. **e** Pie chart representing the percentage of BATF target genes containing either the canonical AP-1 or composite IRF:AP-1 (AICE) motif. **f***t*-SNE plot (left) and violin plot (right) showing the MAGIC gene expression data for the indicated gene. For violin plots, the dots represent mean and lines represent first and third quartile ranges. **g** Representative flow cytometry (left) and geometric mean fluorescence intensity at each cell division (right) for intracellular staining of IRF4 levels versus CTV in LPS-responding B cells 72 h after LPS inoculation. Division 7/8 cells are subdivided into CD138 negative (−) and positive (+). Data represent mean ± SD and statistical significance determined by two-tailed Student’s *t* test. Data represent two independent experiments of nine mice. **h***t*-SNE plot of 6903 *Cd19*^Cre/+^*Irf4*^fl/fl^ (IRF4^–/–^) cells responding to LPS showing the location of six distinct cell clusters. Data represent combined cells from two independent mice. **i***t*-SNE plot of pseudotime ordered cells from (**h**) showing the location of cells from each cluster. **j***t*-SNE plot from (**h**) showing the annotation of each cell with a WT cluster from Fig. 4a. **k***t*-SNE plot from (**h**) showing mean MAGIC expression levels for BATF target genes as in (**c**). **l***t*-SNE plot from (**h**) showing mean MAGIC expression for Leading Edge genes from the indicated gene set (see Fig. 5e). Source data are provided as a Source Data file.

To determine the transcription factors that might be guiding the first differentiation branch point, the SCENIC algorithm^35^, which predicts transcription factor activity, was used to analyze cells in clusters 3 and 7 (Fig. 6a). Correlation of the fold change in expression of transcription factors versus the fold change in that factor’s SCENIC score between clusters revealed a positive correlation between the data. Representing the non-ASC branch, cluster 7 cells were enriched for IRF9 and BACH2 expression and activities. Conversely, cluster 3 cells in the ASC-destined branch were enriched for KLF10, E2F1, and BATF activities. *Batf* was more highly expressed in clusters 3 and 4 compared to 7 and 8 and BATF target genes largely reflected this pattern of activity (Fig. 6b, c). Analysis of BATF target gene expression along pseudotime revealed that the majority were induced early and ultimately repressed and included *Aicda*, *Junb*, *Rela*, *Myc*, and *Batf* itself (Fig. 6d). This is consistent with evidence that BATF-deficient B cells proliferate but cannot class switch due to a failure to induce *Aicda*^36^. Further analysis of the BATF motifs identified by SCENIC above revealed that 35% were canonical AP-1 binding motifs, whereas 65% were AP-1:IRF composite element (AICE) motifs that can be bound by AP-1 family member BATF and IRF4^37^ (Fig. 6e). This suggested that BATF:IRF4 complexes may be important in programming the ASC-destined branch. Our scRNA-seq data revealed that *Irf4* expression peaked in the ASC clusters (5 and 6); however, low expression levels of *Irf4* were observed in early differentiating clusters 3, 4, 7 and 8 (Fig. 6f). Consistent with these data, intracellular staining of B cells responding to LPS in vivo indicated that IRF4 was initially upregulated after division 1 and highly upregulated in division 8+ cells (Fig. 6g).

To explore the role of IRF4 in orchestrating B-cell differentiation trajectories in more detail, we adoptively transferred CTV-labeled *Cd19*^Cre/+^*Irf4*^fl/fl^ (IRF4^–/–^) B cells and performed scRNA-seq on all adoptively transferred cells at 72 h post LPS challenge. *t*-SNE projections revealed IRF4^–/–^ B cells grouped into six distinct clusters, with the majority of the cells assigned to clusters 1 and 2 (Fig. 6h). Consistent with a failure to produce ASC^38^, annotation of the IRF4^–/–^ cells against the bulk RNA-seq data found no match to ex vivo or in vivo ASC (Supplementary Fig. 3). The majority of IRF4^–/–^ cells matched gene expression profiles of naive and actB cells that corresponded to those in division 1, 3, or 5. Pseudotime analysis revealed a more linear trajectory (Fig. 6i and Supplementary Fig. 3). KNN was used to assign each IRF4^–/–^ cell to one of the eight WT scRNA-seq clusters. The majority of cells were assigned to the early clusters (1–3) as well as the two non-ASC branch clusters 7 and 8 (Fig. 6j), further indicating that the IRF4^–/–^ cells failed to initiate the ASC-destined differentiation branch observed in WT B cells. Analysis of BATF signature in the IRF4^–/–^ cells found a lack of BATF activity (Fig. 6k). In addition, IRF4^–/–^ cells displayed little increases in OXPHOS gene expression that was characteristic of the ASC-destined branch (Fig. 6l). Conversely, IRF4^–/–^ cells showed enrichment for inflammatory response genes that marked the non-ASC branch (Fig. 6l). Taken together, these data suggest that IRF4 is necessary to establish the ASC-destined differentiation branch during the actB stage.

### Loss of L-selectin marks B cells destined to become ASC

**Fig. 7:**
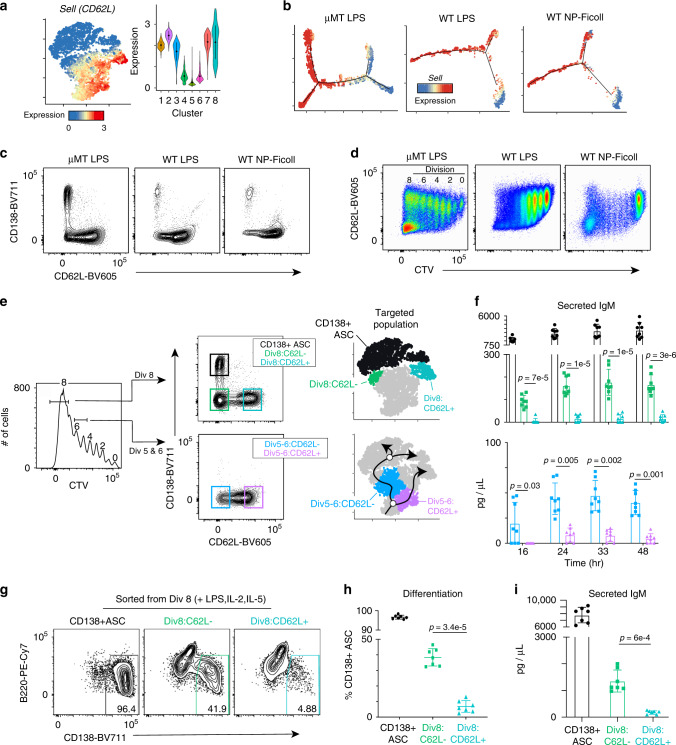
Loss of CD62L (L-selectin) delineates cells on the ASC-destined branch. **a***t*-SNE plot (left) from Fig. 4a and violin plot (right) for each cluster showing the MAGIC gene expression data for *Sell* (CD62L) in WT cells. For violin plots, the dots represent mean and lines represent first and third quartile ranges. **b***Sell* expression projected along pseudotime for each scRNA-seq dataset (see Fig. 5c). **c** Representative flow cytometry plot of CD138 versus CD62L for adoptively transferred cells from μMT hosts challenged with 50 μg of LPS (left), WT hosts challenged with 50 μg of LPS (middle), or WT hosts challenged with 50 μg of NP-Ficoll (right). All plots are 72 h after challenge. **d** Representative flow cytometry plot of CD62L versus CTV for each adoptive transfer setup described in (**c**). The division number is indicated in the left panel. **e** Representative FACS strategy for isolating ASC in clusters 5 and 6 (CD62L^–^CD138^+^ cells), ASC-destined cells in cluster 4 (div8:CD62L^–^CD138^–^), non-ASC-destined cells in cluster 8 (div8:CD62L^+^CD138^–^), ASC-destined cells in cluster 3 (CD62L^–^CD138^–^), and non-ASC-destined cells in cluster 2 (CD62L^+^CD138^–^). Cells were gated on division 5, 6, or 8 and then separated by CD138 and CD62L expression 72 h post LPS challenge. **f** IgM titers in culture over time for the indicated samples from division 8 (top) and divisions 5 and 6 (bottom). **g** Representative flow cytometry plot of B220 versus CD138 expression after 16 h of culture with LPS, IL-2, and IL-5 for the indicated samples. Percent of CD138^+^ ASC is indicated. **h** Percent of CD138^+^ cells and **i** IgM titers in culture for each sample. Data in (**f**, **h**, **i**) represent mean ± SD. Statistical significance in (**f**, **h**, **i**) was determined by paired two-tailed Student’s *t* tests. Data in (**f**) represent two independent experiments with a total of eight mice; data in (**g**–**i**) represent two independent experiments with a total of seven mice. Source data are provided as a Source Data file.

Given the divergent transcriptional programs of the ASC-destined and non-ASC branches, we sought to find markers that would allow separation of cells along either trajectory in vivo. L-selectin (CD62L) or *Sell* is a cell adhesion molecule that facilitates entry of lymphocytes into secondary lymphoid organs from the blood stream^39^ and is expressed as part of the inflammatory response signature that marked the non-ASC branch. Analysis of *Sell* expression in WT cells revealed high levels within clusters 7 and 8 of the non-ASC branch with progressively decreasing levels in the ASC-destined branch (clusters 3–6) (Fig. 7a, b). Similarly, *Sell* expression decreased along the ASC-destined branch in cells responding to LPS and NP-Ficoll in WT hosts. Confirming the scRNA-seq data, flow cytometry revealed that CD62L was ultimately repressed in CD138^+^ ASC in all three systems (Fig. 7c). Analysis of CTV-labeled LPS- or NP-Ficoll-responding B cells showed a gradual bifurcation in CD62L surface expression as B cells divided, with cells appearing to both maintain and lose expression as they progressed through the divisions (Fig. 7d). These data suggest that CD62L could be used to separate B cells committed to either differentiation branch. To test this, we FACS isolated LPS-responding B cells from division 8 that were CD62L^+^CD138^–^ (div8:non-ASC; cluster 8), CD62L^–^CD138^–^ (div8:ASC-destined; cluster 4), and as a positive control, CD138^+^ ASC that were also CD62L^–^ (clusters 5 and 6) (Fig. 7e). Furthermore, we isolated cells from divisions 5 and 6 CD62L^+^CD138^–^ (div5-6:non-ASC; cluster 2) and CD62L^–^CD138^–^ (div5-6:ASC-destined; cluster 3) representing early cells committed to each fate. Each group of cells was incubated in media without further stimulation and CD138^+^ ASC differentiation assessed by monitoring antibody secretion via ELISA over 48 h. Regardless of division, ASC-destined cells secreted significantly more antibody compared to non-ASC cells in the same division across all time points assayed (Fig. 7f). Importantly, because the ASC-destined cells in div5 and 6 secreted IgM, this indicated the div8:non-ASC cells were provided sufficient time to differentiate. Thus, CD62L surface expression can be leveraged to separate actB cells that are destined to become ASC.

Finally, to determine if cells in division 8 on the non-ASC branch could be stimulated to differentiate, we sorted these cells, as well as div8:ASC-destined, and CD138+ ASCs. The cells were cultured for 16 h in the presence of LPS, IL-2, and IL-5 to induce their ex vivo differentiation^40^. ASC formation was assessed by flow cytometry and antibody secretion was monitored by ELISA. Div8:ASC-destined cells readily differentiated into CD138^+^ cells, whereas the div8:non-ASC cells produced tenfold fewer CD138^+^ cells (Fig. 7g, h). Consistent with these findings, Div8:ASC-destined cells secreted significantly more IgM into the culture media compared to div8:non-ASC cells (Fig. 7i). Thus, div8:non-ASC cells do not readily differentiate upon restimulation. Taken together, at early stages of B-cell differentiation, two distinct differentiation trajectories exist with only one leading to ASC.

## Discussion

Using multiple model systems, this study has defined the in vivo relationship between cell division and cellular heterogeneity during B-cell differentiation. B cells responding to T cell-independent type I and II antigens in the absence of cell extrinsic signals required eight cell divisions for CD138^+^ ASC differentiation. Strikingly, scRNA-seq of cells representing all stages of differentiation revealed a divergent actB program, with only a subset of B cells upregulating an IRF4-dependent transcriptional program that led to ASC formation. Cells along this ASC-destined branch downregulated CD62L and formed tenfold more ASC in culture compared to B cells that followed the alternative non-ASC branch. Together, these data define an early cell fate-decision branch point for ASC formation that occurs early in differentiating and dividing actB cells.

Comparing adoptive transfers using μMT, WT, or MYD88^–/–^ mice as hosts and immunizing with LPS revealed that cell extrinsic effects in WT mice can impact the cell division kinetics of B-cell differentiation. In contrast to μMT and MYD88^–/–^ hosts, CD138^+^ ASC differentiation in WT hosts was detected in earlier divisions and indicates that in WT hosts, other LPS-responding cells, such as macrophages and dendritic cells^41^, can influence B-cell differentiation. While these cells are present in μMT hosts, the splenic architecture is abnormal and may impact signals between cells^42^. When the B cell-specific stimulus NP-Ficoll was used, similar cell division/differentiation patterns were observed in both μMT and WT hosts. This indicates that the signaling downstream of toll-like receptor 4 signaling (via LPS) and B-cell receptor signaling (via NP-Ficoll) both initiate a conserved differentiation program that includes the same cell-division constraints. Despite the extrinsic effects observed in the WT hosts stimulated with LPS, the scRNA-seq data revealed a similar cell trajectory in each of the three differentiation models. This suggests that even if extrinsic effects modulate the cell division–differentiation relationship, the molecular reprogramming steps proceed along similar paths. Indeed, ASC-destined and non-ASC branches are observed for both stimuli with similar changes in gene expression, including increases in OXPHOS and MYC-target genes along the ASC-destined branch. Thus in the absence of cell extrinsic modulation, in vivo B-cell differentiation to T-independent type I and II antigens is orchestrated in a manner that requires at least eight cell divisions to fully attain the ASC program.

Our data support the quantal response model of B-cell differentiation to LPS, where the concentration of LPS affects the proportion of cells responding but does not affect the rate of ASC formation within each division^8^. While we cannot rule out that at low doses there is selective activation of B cells that are specific for LPS, our data indicate this all-or-none response to a T cell-independent antigen also occurs in vivo. Lower doses of LPS in vivo impacted the overall magnitude of the B-cell response, with fewer activated/dividing cells observed across the divisions. However, of the cells reaching division 8, the same percentage formed ASC. In contrast to the quantal response model, the kinetics of cell division and differentiation in response to a T cell-dependent antigen follow a distinct graded response model ex vivo, which can be modulated by amounts of CD40L^8^. In vivo, T cell-dependent responses are further complicated due to the complex dynamics of the germinal center reaction, cyclic reentry, and selection pressure for high affinity clones^43,44^. Using type I and type II T cell-independent stimuli, our scRNA-seq data demonstrate that B cells progress down similar ASC differentiation paths in response to both BCR and TLR signals. Thus, these data confirm key aspects of ex vivo differentiation models to TI antigens and add new cell division parameters to help predict adaptive immune responses in vivo.

Here, transcriptional heterogeneity was observed both within cells at the same division and among cells at distinct differentiation stages. Despite the observed heterogeneity, a consistent percentage of responding B cells forms ASC in an experimentally repeatable manner. Our data suggest that IRF4, in tandem with BATF at AICE DNA-binding elements, enforces early programming changes that drive a subset of B cells toward the ASC fate. The stochastic differentiation model predicts cell fate outcomes based on a probability distribution across the population of responding cells^45^ and can be applied here to the bifurcation of ASC-destined versus non-ASC cells observed in the scRNA-seq data. In fact, the concentration of IRF4 is critically important for determining B-cell fate^46–48^. Thus, the probability of inducing sufficient IRF4 expression to initiate the ASC-destined branch may be the same for each cell, with higher doses of LPS resulting in a greater number of cells being stimulated sufficiently to progress toward ASC.

One of the defining features of the ASC-destined branch was the loss of CD62L (L-selectin) expression. Thus, CD62L expression was used as a surface marker to distinguish subsets of actB cells along divergent differentiation trajectories. CD62L is an adhesion molecule that mediates leukocyte migration to secondary lymphoid tissues, and shedding of CD62L allows leukocytes to be redirected to sites of infection^39^. In addition to its role as an adhesion molecule, CD62L also plays a role in triggering cell signaling events^49^. In our system, CD62L expression distinguished ASC-destined cells as early as division 5. In fact, the small number of ELISPOTs observed in division 8 CD138^–^ cells may be due to cells that are continuing to differentiate during the ex vivo assay. The function of reduced CD62L could allow ASC-destined cells to remain in circulation where they could eventually home to the bone marrow for long-term survival. While the full role that CD62L plays in B-cell biology is not fully understood, our data demonstrate that it is a useful marker to distinguish actB cells destined to differentiate to ASC.

Trajectory analysis of B cells differentiating to LPS in μMT hosts identified a second bifurcation event following differentiation that split differentiated ASC into two groups. Cells assigned to ASC clusters 5 and 6 displayed all the hallmarks of ASC, including high expression of *Xbp1*, a transcription factor required for initiating the unfolded protein response^50,51^. This indicates that cells in both clusters had initiated the ASC transcriptional program to counter ER stress. However, *Atf6*, an ER stress sensor was highly expressed in cluster 6. This suggests that these cells may be under distinct ER stress compared to ASC in cluster 5. Support for this concept comes from data showing that in response to LPS stimulation, targets of ATF6 in the I.29 micro(+) B-cell line were upregulated later than other aspects of the unfolded protein response^52^. Although the same ASC bifurcation event was not observed from scRNA-seq when using WT hosts, this may be due to the timing in which the cells were harvested or limited by the numbers of ASCs generated and sequenced. Differences in the ability to regulate ER stress may provide a means to select for ASC that can adapt to the stress of Ig production and secretion.

Together, these data provide insight into the heterogeneity and cell division kinetics of B-cell differentiation in vivo and highlight an important IRF4-dependent bifurcation event that occurs early during actB reprogramming, with only a subset of B cells leading to ASC. This ASC-destined branch required an alignment of cellular proliferation and metabolic reprogramming events that enabled ASC formation and their ability to withstand the stress of high antibody production and secretion. The alternative non-ASC branch contained cells that were responding to inflammatory stimuli and may ultimately have a role in shaping immune responses. Ultimately, controlling which pathway cells follow could help in the therapeutic control of B cell-mediated immunity.

## Methods

### Mice and adoptive transfers

C57BL/6J (JAX; 000664), CD45.1 (JAX; 002014), *Cd19*^*C*re^ (JAX; 006785)^53^, *Irf4*^fl/fl^ (JAX; 009380)^38^, *Prdm1*^fl/fl^ (JAX; 008100)^54^, MYD88^–/–^ (JAX; 009088)^27^, and CD45.2 μMT mice (JAX; 002288)^42^ were purchased from The Jackson Laboratory and bred on site. CD45.2 μMT were bred onto the CD45.1 background to obtain homozygous CD45.1 μMT mice. Mice used in experiments were between 8 and 12 weeks of age, were age/gender matched, and all genders were equally represented throughout the experiments. For adoptive transfers/division assays, splenic CD43^–^ B cells were isolated using the Miltenyi B-cell Isolation Kit (Miltenyi; 130-090-862), which yielded equivalent proportions of B cells for all input populations in this study (Supplementary Fig. 4). Isolated B cells were stained with CTV per the manufacturer’s protocol, and 15 × 10^6^ CTV-labeled B cells were transferred to a disparate congenic μMT, MYD88^–/–^, or WT mouse (CD45.1 or CD45.2) intravenously. At 24 h post transfer, mice were inoculated with the indicated dose of LPS (Enzo Life Sciences; ALX-581-008) or 50 μg NP-Ficoll (Biosearch Technologies; F-1420-10) intravenously in 100 μl. Animals were randomly placed in experimental groups, and independent replicates of three or more were used for all phenotyping experiments. Experimental mice were euthanized via carbon dioxide asphyxiation in accordance with AVMA Guidelines, 2020 edition. Animals were housed in specific pathogen-free caging with 12 h light/dark cycles by the Emory Division of Animal Resources and all procedures were approved by the Emory Institutional Animal Care and Use Committee.

### Flow cytometry and cell sorting

For antibody staining, cells were resuspended at a concentration of 1 × 10^6^/100 μl in FACS buffer (1X PBS, 2 mM EDTA, and 1% BSA), stained with Fc Block (BD; 553141) for 15 min, antibody–fluorophore conjugates for 30 min, and then washed with 10 volumes of FACS buffer. For flow cytometry analyses all cells were fixed using 1% paraformaldehyde. For enrichment of adoptively transferred CD45.2 or CD45.1 congenically marked cells, splenocytes were stained with CD45.2-PE or CD45.1-PE, respectively, followed by immunomagnetic enrichment using anti-PE beads (Miltenyi, 130-097-054). For analysis of adoptive transfers into WT hosts, all cells were enriched prior to flow cytometry analysis. For scRNA-seq experiments from µMT hosts, all transferred cells were FACS isolated. For scRNA-seq experiments from WT hosts, cells in divisions 1–8 and division 0 were FACS isolated separately and mixed to achieve a final ratio of 95% responding (dividing cells, divisions 1–8) and 5% non-responding (undivided, division 0) cells (Supplementary Fig. 1). The following specific antibody–fluorochrome conjugates, with specific clones indicated in parentheses, and stains were used (Supplementary Data 1): Tonbo Biosciences, Inc.: CD45.1-FITC (A20), CD45.1-APC-Cy7 (A20), CD45.2-PE (104), and CD45.2-PerCPCy5.5 (104); BioLegend, Inc.: B220-PE-Cy7 (RA3-6B2), B220-A700 (RA3-6B2), CD138-APC (281-2), CD62L-PerCPCy5.5 (MEL-14), CD62L-BV605 (MEL-14), CD45.1-PE (A20), CD45.1-APC (A20), CD45.2-PE-Cy7 (104), CD45.2-FITC (104), CD45.2-APC (104), CD11b-APC-Cy7 (M1/70), F4/80-APC-Cy7 (BM8), CD90.2-APC-Cy7 (30H12), Zombie Yellow Fixable Viability Kit (Cat. #423104), and Zombie NIR Fixable Viability Kit (Cat. #423106); BD Biosciences, Inc.: CD138-BV711 (281-2); ThermoFisher, Inc.: IRF4-PerCPeFluor710 (3E4); Life Technologies, Inc.: CTV (Cat. #C34557). For intracellular staining of IRF4, the FIX & PERM Cell Permeabilization Kit (ThermoFisher; GAS003) was used per the manufacturer’s protocol. For all flow cytometry analysis, the following gating strategy was used (Supplementary Fig. 5): lymphocytes based on SSC-A and FSC-A, sc based on FSC-H and FSC-W, live cells based on exclusion of Zombie Yellow or Zombie NIR Fixable Viability Kit, and the markers CD11b^–^F4/80^–^CD90.2^–^ to remove non-B cell lineage cells. All flow cytometry data were collected on a LSR II or LSRFortessa (BD Biosciences) and analyzed using FlowJo v9.9.5 or FlowJo v10.6.2. Cell sorting was performed at the Emory Flow Cytometry Core or the Pediatrics and Winship Advanced Flow Cytometry Core using a FACSAria II (BD Biosciences) and BD FACSDiva software v8.0 (BD Biosciences).

### ELISPOT

ELISPOT plates (Millipore; MAHAS4510) were coated with 1 μg of capture antibody (SouthernBiotech; 1010-01) for 12 h, washed with PBS three times, and then blocked with B-cell media (RPMI 1640 supplemented with 10% heat-inactivated FBS, 0.05 mM 2-BME, 1X nonessential amino acids, 1X penicillin/streptomycin, 10 mM HEPES, and 1 mM sodium pyruvate) for >1 h. B cells were sorted directly into B-cell media and resuspended to desired volume/cell numbers for plating. A 1:2 dilutions series was performed, and 70 μl of the diluted cells was added to each well and incubated (37 °C, 5% CO_2_) for 5 h. Plates were then washed five times with PBS-T (1X PBS, 0.05% Tween-20) and secondary antibody (SouthernBiotech; Goat anti-mouse Ig, 1010-04) conjugated to alkaline phosphatase was diluted 1:500 in PBS and 100 μl added for 1 h at room temperature. Plates were then washed five times in PBS-T and 5-Bromo-4-chloro-3-indolyl phosphate reagent (SouthernBiotech; 0302-01) was added to each well for 15 min to allow spots to develop. Finally, plates were washed four times in H_2_O and allowed to dry completely before imaging. All samples and all dilutions were performed in technical duplicates. Spots were imaged using a ImmunoSpot S6 ULTIMATE Analyzer (Cellular Technology Limited) and quantified using ImmunoSpot software v5.0.9.21.

### Ex vivo B-cell differentiation

Cells were sorted and resuspended in B-cell media containing 20 mg/ml *Escherichia coli* O111:B4 derived LPS (Sigma-Aldrich; L2630), 5 ng/ml IL-5 (eBioscience; 14-8051), and 20 ng/ml IL-2 (eBioscience; 14-8021).

### ELISA

Flat-bottomed ELISA plates (Sigma-Aldrich; M9410) were coated with goat anti-mouse Ig (Southern Biotechnology; 1010-01) overnight at 4 °C. Plates were then washed (1X PBS containing 0.05% Tween-20), blocked with 1% nonfat dry milk, and incubated with a series of diluted samples or purified mouse IgM (Southern Biotechnology; 5300-01B). Plates were then washed, incubated with 1:1000 diluted HRP-conjugated goat anti-mouse IgM (Southern Biotechnology; 1021-05), washed, and incubated with tetramethylbenzidine ELISA peroxidase substrate (Rockland; TMBE-1000) for 20 min at room temperature (50 μl/well). 50 μl of 0.2 M sulfuric acid was applied to each well to quench the reaction. All incubations were performed for 1 h at room temperature unless stated otherwise. Plates were read using a BioTek ELx800 Absorbance Microplate Reader using BioTek Gen5 Microplate Reader Software v3.02.

### Bulk RNA sequencing

For each sample, 1000 cells were sorted into 300 μl of RLT buffer (Qiagen; 79216) containing 1% BME. RNA was extracted using the Quick-RNA Microprep kit (Zymo Research; R1050) and all purified RNA was used as input for the SMART-seq v4 cDNA synthesis kit (Takara; 634894) with 12 cycles of PCR amplification. 400 pg of cDNA was used as input for the NexteraXT kit (Illumina) using 12 cycles of PCR amplification. Final libraries were quantitated by qPCR, size distributions determined by bioanalyzer, pooled at equimolar ratios, and sequenced at the New York University Genome Technology Center on a HiSeq3000 using a PE50 run.

### Bulk RNA sequencing analysis

Raw sequencing reads were mapped to the mm9 genome using TopHat2 v2.0.13^55^ with the UCSC KnownGene reference transcriptome^56^ and the ERCC spike-in RNA controls^57^. Duplicate reads were marked by PICARD v1.127 (http://broadinstitute.github.io/picard/) and removed from downstream analysis. Reads overlapping exons of each ENTREZ gene and the ERCC transcripts were summarized and normalized to fragments per kilobase per million using GenomicRanges v1.34^58^ and custom R v3.5.2 scripts. Genes with at least three reads per million in all samples for at least one group were considered detected and resulted in 10,532 expressed genes. All detected genes were used as input for edgeR v3.24.3^59^ and genes with an absolute log_2_ fold change > 1 and Benjamini–Hochberg FDR < 0.05 were considered significantly differentially expressed. PCA was performed using vegan v2.5.5 and the indicated *z*-score normalized gene list. mRNA molecules per cell normalization was computed as follows^16^ with normalized mRNA content representing the sum total of all mRNA molecules in each sample:

$$\frac{{\rm{mRNA}}_{{\rm{gene}}\,{\rm{A}}}}{{\rm{Cell}}} = \frac{{\rm{FPKM}}_{{\rm{gene}}\,{\rm{A}}}}{ {\rm{No. of}}\,{\rm{cells}}} \times \frac{\sum {\rm{molecules}}_{{\rm{ERCC}}}}{\sum {\rm{FPKM}}_{{\rm{ERCC}} }}.$$

### Single-cell RNA sequencing and data processing

scRNA sequencing was performed using the 5′ scRNA-seq platform (10X Genomics). For each sample an estimated 17,400 cells were used as input for GEM generation and libraries prepared using the recommended protocol. Illumina bcl files were processed and mapped to the mm10 genome using CellRanger v2.1.1 and each biological replicate aggregated for downstream analysis using the “aggr” function in CellRanger with the default parameters. Aggregated data were analyzed using Monocle2 v2.9.0^31^. For *t*-SNE dimension reduction the following parameters were used: max_components = 2, norm_method = “log,” num_dim = 10, perplexity = 30. Assignment of cells to clusters was performed using the default rho and delta thresholds determined by the “plot_rho_delta” function. To determine DEG between clusters the “differentialGeneTest” function with the “fullModelFormulaStr” option was used. The top 1000 DEG ranked by FDR *q* value was used in the sc trajectory analysis with the “orderCells” function with default parameters and dimensional reduction performed using the “DDRTree” function.

### MAGIC transformation of UMI transcript counts

For MAGIC normalization^60^ the Rmagic v1.3.0R/Bioconductor package was used. The UMI gene expression matrix was exported from the Monocle CellDataSet object using the “exprs” function. Genes that were expressed in <10 cells and cells with <1000 total UMI were removed from the analysis. The data were normalized for library size and square root transformed and the resulting matrix was used as input for the “magic” function using the “genes = all_genes” parameter.

### SCENIC transcription factor activity prediction

The SCENIC v1.1.1R/Bioconductor package^35^ was used to predict transcription factor activity in each cell using the mm10 version of the cisTarget database (https://resources.aertslab.org/cistarget/). The UMI gene expression matrix was filtered using the “geneFiltering” function with the parameters minCountsPerGene = 10 and minSamples = 9 and the resulting matrix log_2_ transformed. Next, all subsequent steps of Genie3 and SCENIC were run with the default parameters. The resulting output file “regulonTargetsInfo.tsv” file was used to identify transcription factor target genes. The correlation of SCENIC activity score and expression was calculated using the “cor” function with the Pearson method in R.

### KNN classification of single cells with bulk RNA-seq data

KNN classification was performed using the FNN v1.1.2.1R package. The UMI gene expression matrix was merged with reads per million normalized bulk RNA-seq datasets using gene symbol as unique identifiers. The combined data were quantile normalized using “normalize.quantiles” function in the preprocessCore v1.40.0 package. Next, to assign bulk RNA-seq to sc, the “knn” function was run using the bulk RNA-seq samples as a training set, sc as the testing set, and the K was set to the number of replicates in bulk the RNA-seq experiments. The resulting cell assignments were appended to the pData table associated with the Monocle CellDataSet object. For assignment of IRF4^−/−^ cells to WT scRNA-seq clusters the UMI matrices containing each cell were merged using the gene symbol and quantile normalized. The “knn” function was run using the WT cells as the training set, the IRF4^−/−^ cells as the testing set, and the K was set to 11. The IRF4^−/−^ cells were then annotated with the WT cluster of the cell they were assigned.

### scRNA-seq data display

For all data display, the MAGIC^60^ transformed gene expression data were used. For GSEA^32^ between clusters, all detected genes were ranked by multiplying the *P* value derived from the Monocle “differentialGeneTest” function by the sign of the fold change (i.e., positive or negative) between the two clusters. The resulting list was used in a GSEA PreRanked analysis. All indicated gene sets were derived from the Molecular Signatures Database^32^ except the ER chaperones, co-chaperones, and folding enzymes gene set, which was previously described^34^. For gene set activity plots, the mean expression cubed of all leading edge genes was computed and visualized as a projection on the *t*-SNE plot. For BATF transcription factor activity, the mean expression cubed of all target genes predicted by SCENIC was computed and visualized as above. All other data display was performed using custom R/Bioconductor scripts that are available upon request.

### Statistics

All statistical analyses were performed with GraphPad Prism v6.0c or v8.4.1, Microsoft Excel v14.5.7 or v16.36, and R/Bioconductor v3.5.2. The exact statistical test, number of tails, group size, and number of experimental repeats for each figure are detailed in the legend. *P* values or where indicated multiple hypothesis testing corrected *P* values < 0.05 were considered significant. For differential gene expression a combination of fold change and false-discovery rate corrected *P* values was used to determine significance.

### Reporting summary

Further information on research design is available in the Nature Research Reporting Summary linked to this article.

## Supplementary information

Supplementary Information

Reporting Summary

Description of Additional Supplementary Files

Supplementary Data 1

## Data Availability

All sequencing data have been deposited in NCBI Gene Expression Omnibus (GEO) under the accession codes GSE136275 (Bulk RNA-seq) and GSE136376 (scRNA-seq). Source data are provided with this paper.

## Source data

Source Data

## Code availability

Code and data processing scripts are available from the corresponding author upon request and at https://github.com/cdschar/scRNAseq_NatComm2020. Source data are provided with this paper.
