# Supplementary material for: Antibody-secreting cell destiny emerges during the initial stages of B-cell activation
Source: Nat Commun. 2020 Aug 10;11:3989. doi: 10.1038/s41467-020-17798-x (PMC7417592; doi:10.1038/s41467-020-17798-x)
Supplement: Supplementary file 1 — Supplementary Information [file 41467_2020_17798_MOESM1_ESM.pdf]

Antibody-secreting cell destiny emerges during the initial stages of B cell activation

Christopher D. Scharer[1,2], Dillon G. Patterson[1,2], Tian Mi[1], Madeline J. Price[1], Sakeenah L. Hicks[1], and Jeremy M. Boss[1]

[1]Department of Microbiology and Immunology and the Emory Vaccine Center, Emory University School of Medicine, Atlanta, GA 30322, USA

[2]These authors contributed equally

Contact: jmboss@emory.edu

SUPPLEMENTARY FIGURES

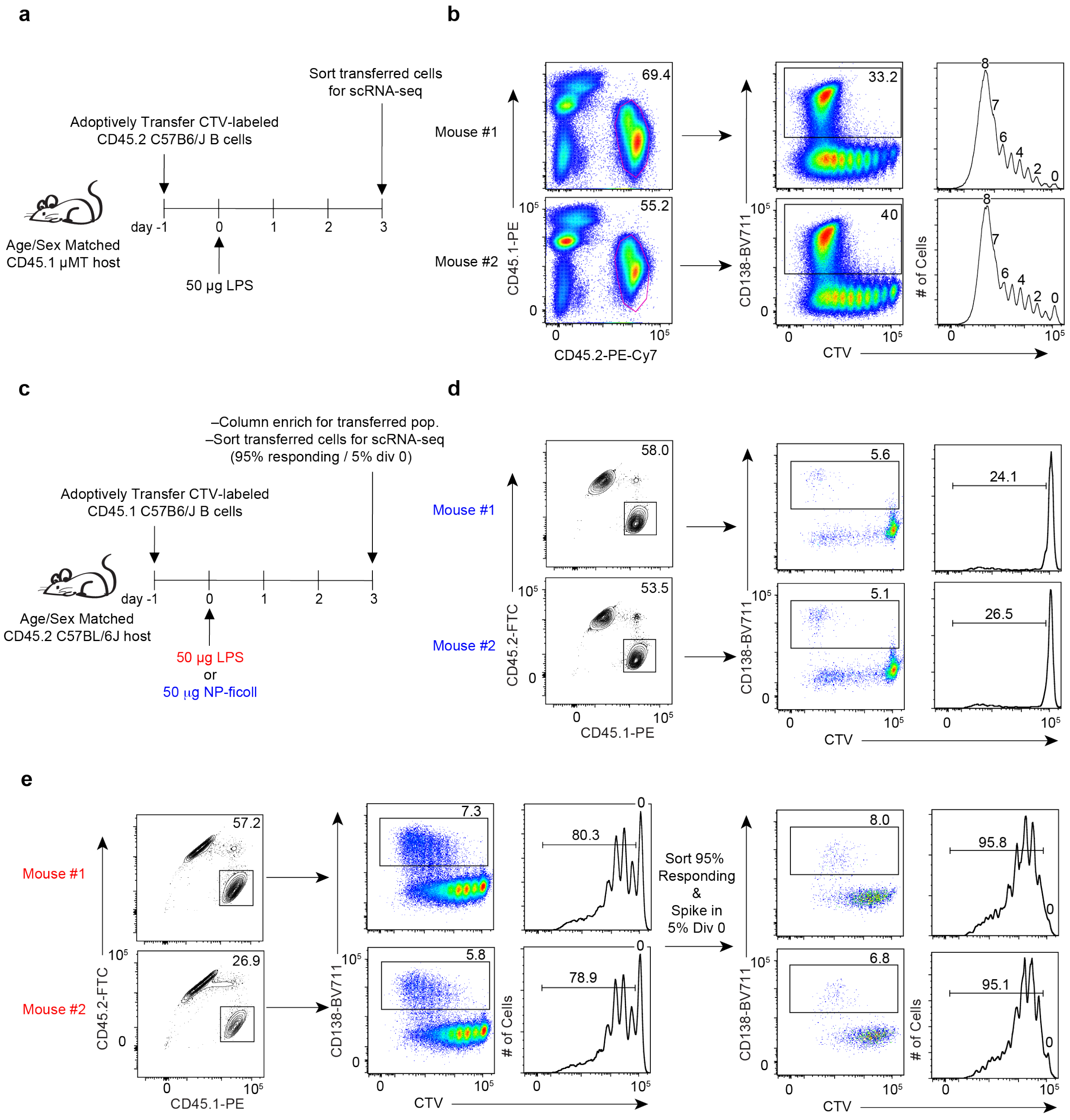

Supplementary Figure 1. Summary of adoptive transfers used to generate scRNA-seq of LPS or NP-Ficoll responding wild-type B cells.

(**a**) Schematic of experimental design for μMT hosts challenged with LPS. (**b**) Flow cytometry plot of CD45.1 versus CD45.2 with percentage of CD45.2^{+} cells displayed (left), CD138 versus CTV with percentage of CD138^{+} cells shown (middle), and CTV histograms with division numbers

indicated (right) for each mouse used to generate scRNA-seq data (see **Fig. 4**). (**c**) Schematic of experimental design for WT hosts challenged with LPS or NP-Ficoll. (**d**) For NP-Ficoll, flow cytometry plot of CD45.2 versus CD45.1 with percentage of CD45.1$^+$ cells displayed (left), CD138 versus CTV with percentage of CD138$^+$ cells shown (middle), and CTV histograms with the frequency of cells that have responded indicated (right) for each mouse. A mixture of 95% responding cells and 5% division 0 cells were used to generate scRNA-seq data (final input not shown; see **e** for example). (**e**) For LPS, flow cytometry plot of CD45.2 versus CD45.1 with percentage of CD45.1$^+$ cells displayed (left), CD138 versus CTV with percentage of CD138$^+$ cells shown (middle-left), and CTV histograms with the frequency of responding cells indicated (middle-right) for each mouse. A mixture of 95% responding cells and 5% division 0 cells were used to generate scRNA-seq data; final input for scRNA-seq with the same gates/frequencies shown as the middle panel (right).

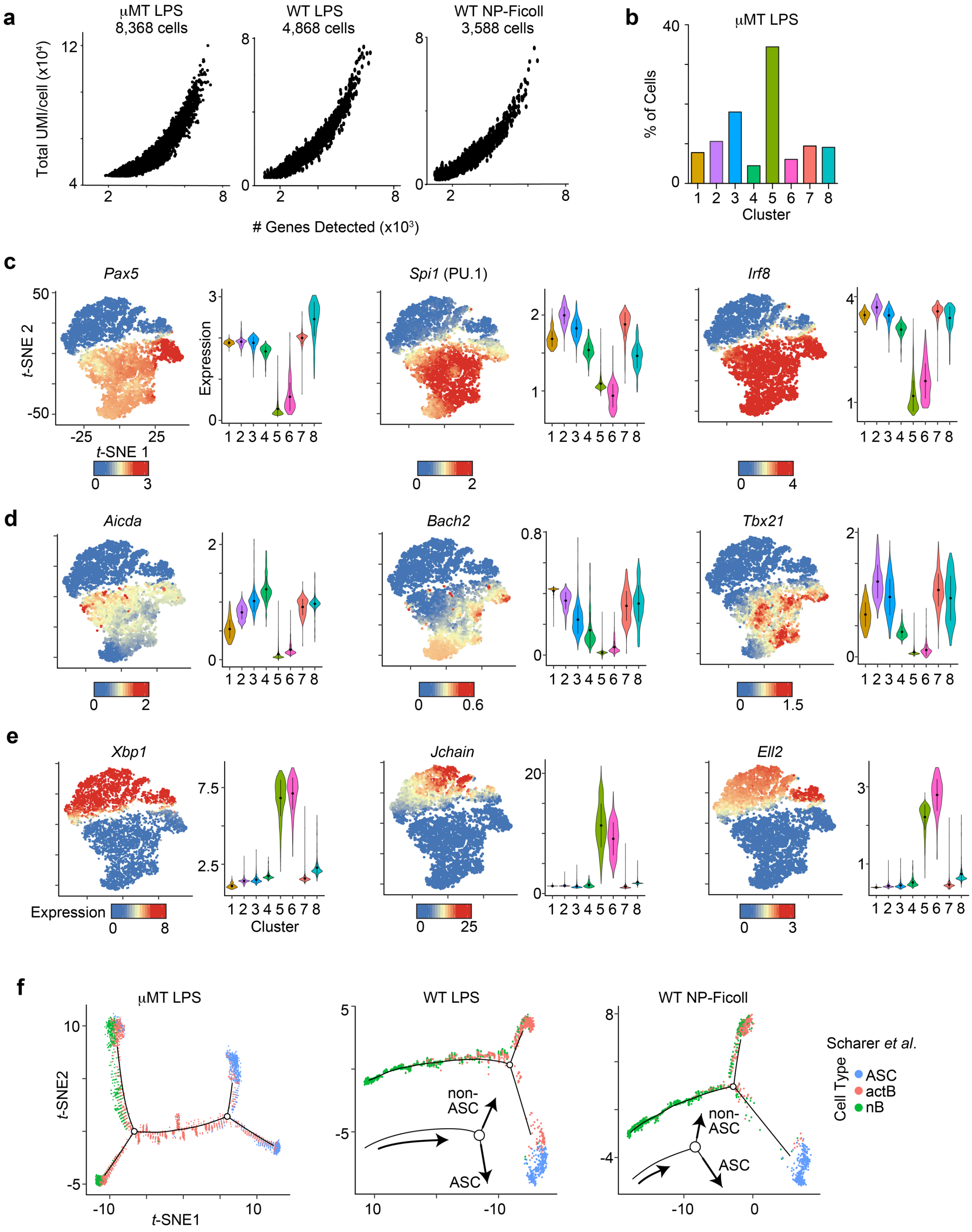

a
μMT LPS
8,368 cells
WT LPS
4,868 cells
WT NP-Ficoll
3,588 cells
Total UMI/cell (x10⁴)
Genes Detected (x10³)
b
μMT LPS
% of Cells
Cluster
c
Pax5
Spi1 (PU.1)
Irf8
t-SNE 2
t-SNE 1
Expression
d
Aicda
Bach2
Tbx21
e
Xbp1
Jchain
Ell2
Expression
Cluster
f
μMT LPS
WT LPS
WT NP-Ficoll
t-SNE2
t-SNE1
non-ASC
ASC
Scharer et al.
Cell Type
ASC
actB
nB

Supplementary Figure 2. scRNA-seq of transferred CTV-labeled cells captures a continuum of responding B cells at all stages of differentiation.

(**a**) Scatterplot of unique molecular identifiers (UMI) versus number of genes detected per cell for each of the three scRNA-seq datasets. (**b**) Bar plot of the frequency of cells assigned to each cluster for the μMT LPS scRNA-seq dataset. *t*-SNE plot (left) from **Fig. 4a** and violin plots (right) for each cluster showing the MAGIC gene expression data for the indicated genes representing (**c**) B cells, (**d**) activated B cells, and (**e**) ASC. For violin plots, the dots represent mean and lines represent 1st and 3rd quartile ranges. (**f**) Pseudotime *t*-SNE plot from μMT LPS (left), WT LPS (middle), or WT NP-Ficoll (right) showing cells annotated with naïve B cells (nB) and *ex vivo* differentiated activated B cells (actB) and ASC from Scharer *et al*[14]. Circles denote pseudotime branch points. See also **Fig. 5**. Source data are provided as a Source Data file.

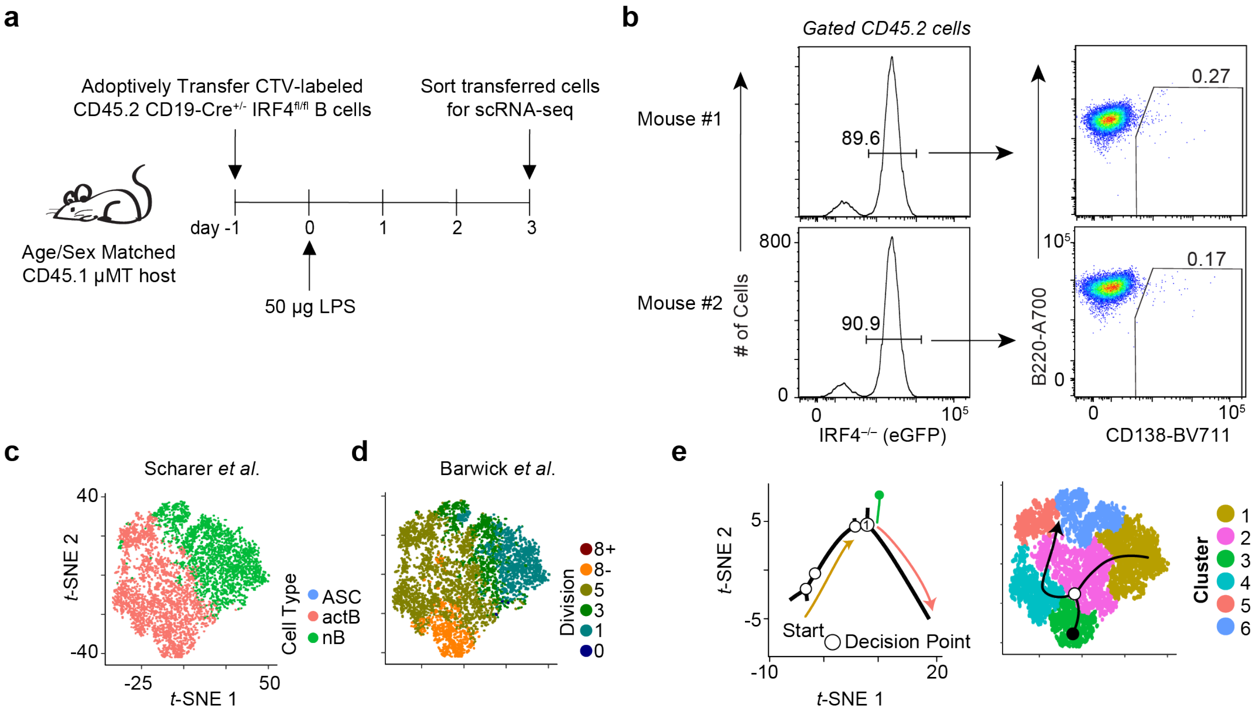

Supplementary Figure 3. scRNA-seq of bulk transferred CTV-labeled IRF4-deficient B cells reveals a critical role for IRF4 in establishing the ASC-destined branch.

(**a**) Schematic of experimental design. (**b**) Flow cytometry plot of histograms of eGFP, which marks IRF4-null cells[40] (left) and B220 versus CD138 (right) are shown after previously gating CD45.2^{+} transferred cells for each mouse used to generate scRNA-seq data (see **Fig. 6h**). The percentage of eGFP (left) and CD138^{+} (right) cells are indicated. (**c**) *t*-SNE plot from **Fig. 6h** showing the annotation of each cell with naïve B cells (nB) and *ex vivo* differentiated activated B cells (actB) and ASC from Scharer *et al*[14]. (**d**) *t*-SNE plot from **Fig. 6h** showing the annotation of each cell with division sorted LPS-responding B cells from Barwick *et al*[16]. (**e**) Schematic showing the pseudotime order of cells from **Fig. 6i** (left) and from the *t*-SNE plot from **Fig. 6h** (right).

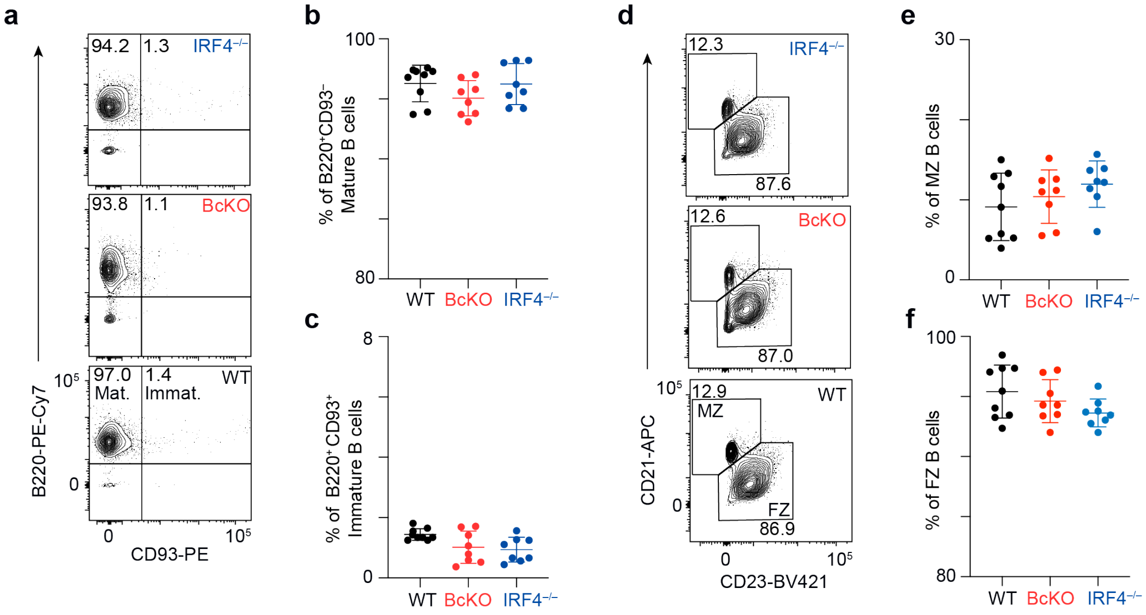

Supplementary Figure 4. Equivalent CD43^{-} B cell populations were enriched from each mouse strain.

(**a**) Representative flow cytometry plots of B220 versus CD93 following B cell isolation for IRF4$^{-/-}$ (top), BcKO (middle), and WT mice (bottom). Quadrant I shows the frequency of immature B cells (B220^{+}CD93^{+}), while quandrant II shows the frequency of mature B cells (B220^{+}CD93^{-}). Data summary of the frequency of mature (**b**) and immature (**c**) B cells for all replicates. (**d**) Representative flow cytometry plots of CD21 versus CD23 following B cell isolation for IRF4$^{-/-}$ (top), BcKO (middle), and WT mice (bottom). Frequency of marginal zone (MZ; CD21^{+}CD23^{-}) and follicular zone (FZ; CD21^{-}CD23^{+}) B cells are shown. Quantification of the frequency of MZ (**e**) and FZ (**f**) B cells for all replicates. Data in **b**, **c**, **e**, **f** represent mean ± SD. Data were derived from two independent experiments of 8 total IRF4$^{-/-}$ and BcKO mice and 9 total WT mice. Source data are provided as a Source Data file.

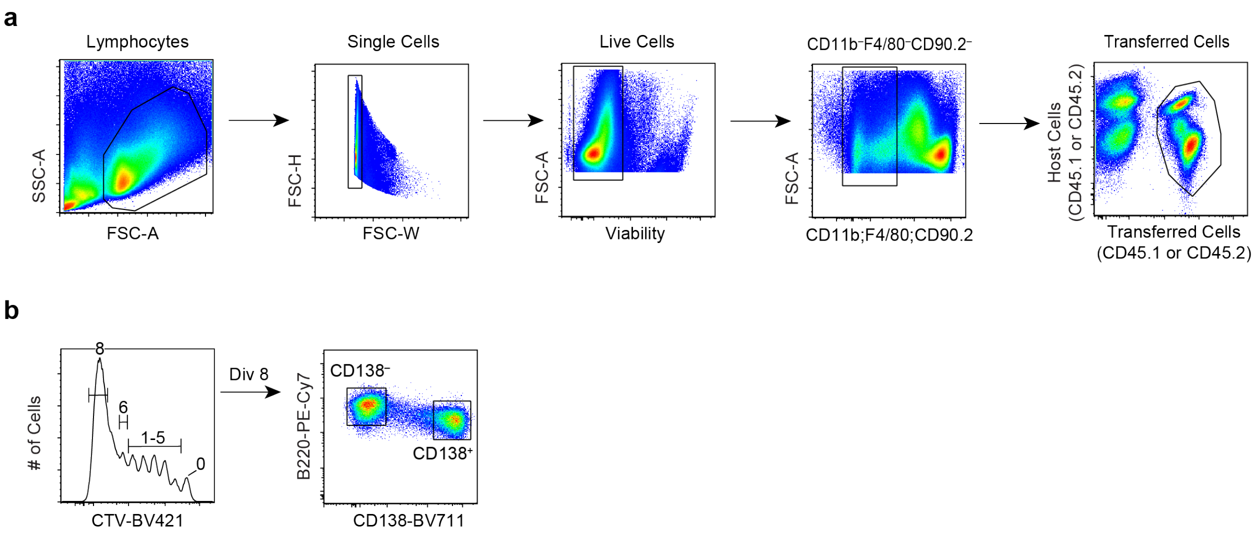

Supplementary Figure 5. General gating strategies used for adoptive transfer analysis and cell sorting.

(**a**) General gating strategy used to analyze all adoptively transferred cells with the purpose of each gate indicated at the top of each panel. Once live, single transferred cells were distinguished from host cells, other parameters, such as cell division and differentiation, could be assessed as in **Fig.1a-g**, **Fig. 2**, **Fig. 3a**, **Fig. 6g**, and **Fig. 7c,d**. These same gating strategies were applied before sorting transferred cells in **Fig. 4**, **Fig. 5**, and **Fig. 6** for single cell RNA-sequencing (detailed in **Supplementary Fig. 1** and **Supplementary Fig. 3**) or for sorting cells along the ASC-destined and non-ASC branch (detailed in **Fig. 7e-i**). (**b**) Gating strategy to sort responding B cells in discrete divisions for ELISPOTs presented on **Fig.1h,i**. Transferred cells were gated before examining cell division via CTV using the gating strategy outlined in **a**. Division 8 cells were sorted into distinct populations based on CD138 expression. The same strategy was used to sort Ctrl and BcKO cells presented in **Fig. 3** with the appropriate divisions gated.
