# Supplementary material for: Antibody-secreting cell destiny emerges during the initial stages of B-cell activation
Source: Nat Commun. 2020 Aug 10;11:3989. doi: 10.1038/s41467-020-17798-x (PMC7417592; doi:10.1038/s41467-020-17798-x)
Supplement: Supplementary file 2 — Reporting Summary [file 41467_2020_17798_MOESM2_ESM.pdf]

nature research

Corresponding author(s): Boss, Jeremy M.
Last updated by author(s): 07/13/2020

Reporting Summary

Nature Research wishes to improve the reproducibility of the work that we publish. This form provides structure for consistency and transparency in reporting. For further information on Nature Research policies, see Authors & Referees and the Editorial Policy Checklist .

Statistics

For all statistical analyses, confirm that the following items are present in the figure legend, table legend, main text, or Methods section.

n/a	Confirmed	
☐	☒	The exact sample size (*n*) for each experimental group/condition, given as a discrete number and unit of measurement
☐	☒	A statement on whether measurements were taken from distinct samples or whether the same sample was measured repeatedly
☐	☒	The statistical test(s) used AND whether they are one- or two-sided<br>*Only common tests should be described solely by name; describe more complex techniques in the Methods section.*
☒	☐	A description of all covariates tested
☐	☒	A description of any assumptions or corrections, such as tests of normality and adjustment for multiple comparisons
☐	☒	A full description of the statistical parameters including central tendency (e.g. means) or other basic estimates (e.g. regression coefficient) AND variation (e.g. standard deviation) or associated estimates of uncertainty (e.g. confidence intervals)
☐	☒	For null hypothesis testing, the test statistic (e.g. *F*, *t*, *r*) with confidence intervals, effect sizes, degrees of freedom and *P* value noted<br>*Give P values as exact values whenever suitable.*
☒	☐	For Bayesian analysis, information on the choice of priors and Markov chain Monte Carlo settings
☒	☐	For hierarchical and complex designs, identification of the appropriate level for tests and full reporting of outcomes
☐	☒	Estimates of effect sizes (e.g. Cohen's *d*, Pearson's *r*), indicating how they were calculated

Our web collection on statistics for biologists contains articles on many of the points above.

Software and code

Policy information about availability of computer code

Data collection	Flow cytometry data were collected with BD FACSDiva v8.0, ELISA data were collected with a BioTek Gen5 Microplate Reader using BioTek Gen5 Microplate Reader Software v3.02, ELISPOT images were collected using a ImmunoSpot S6 ULTIMATE Analyzer (Cellular Technology Limited; CTL) and quantified using ImmunoSpot software (ImmunoSpot v5.0.9.21)
Data analysis	FlowJo v9.9.5 or v10.6.2, GraphPad Prism v6.0c or v8.4.1, Microsoft Excel v14.5.7 or v16.36, ImmunoSpot v5.0.9.21, BioTek Gen5 Microplate Reader Software v3.02, TopHat2 v2.0.13, PICARD v1.127, GenomicRanges v1.34, R v3.5.2, edgeR v3.24.3, vegan v2.5.5, CellRanger v2.1.1, Monocle2 v2.9.0, Rmagic v1.3.0, SCENIC v1.1.1, FNN v1.1.2.1, GSEA v1

For manuscripts utilizing custom algorithms or software that are central to the research but not yet described in published literature, software must be made available to editors/reviewers. We strongly encourage code deposition in a community repository (e.g. GitHub). See the Nature Research guidelines for submitting code & software for further information.

Data

Policy information about availability of data

All manuscripts must include a data availability statement. This statement should provide the following information, where applicable:

- Accession codes, unique identifiers, or web links for publicly available datasets
- A list of figures that have associated raw data
- A description of any restrictions on data availability

All sequencing data has been deposited in NCBI Gene Expression Omnibus (GEO) under the accession codes GSE136275 (Bulk RNA-seq, [https://www.ncbi.nlm.nih.gov/geo/query/acc.cgi?acc=GSE136275]) and GSE136376 (scRNA-seq, [https://www.ncbi.nlm.nih.gov/geo/query/acc.cgi?acc=GSE136376]).

Field-specific reporting

Please select the one below that is the best fit for your research. If you are not sure, read the appropriate sections before making your selection.

[x] Life sciences   [ ] Behavioural & social sciences   [ ] Ecological, evolutionary & environmental sciences

For a reference copy of the document with all sections, see nature.com/documents/nr-reporting-summary-flat.pdf

Life sciences study design

All studies must disclose on these points even when the disclosure is negative.

Sample size	Sample size was determined based on the number of mice per group that show robustness and reproducibilty of the phenotypes. All experiments were independently replicated 2 or more times with at least 3 mice per group. All genomics were performed on 2 or more independent animals as recommended by the ENCODE Consortium.
Data exclusions	No data were excluded from the analysis.
Replication	All mouse phenotyping was successfully replicated across multiple cohorts of mice performed at independent times. The BLIMP-1 RNA-seq data was performed once and matches with previously described data. The uMT LPS scRNA-seq data and branching trajectory was replicated in two independent scRNA-seq experiments - WT LPS and NP-Ficoll datasets. The expression of CD62L was validated from the scRNA-seq data in independent cohorts of mice. All attempts at replication were successful.
Randomization	For all mouse experiments the distribution of age and gender was matched between experimental and control groups. For phenotyping of wild-type mouse responses to LPS, animals were randomly placed in experimental groups.
Blinding	There was no risk of bias in this study from knowing the sample details, so blinding was not relevant. Our experiments were primarily based on flow cytometric analysis, which is not subject to the same potential biases as experiments with more subjective outcomes.

Reporting for specific materials, systems and methods

We require information from authors about some types of materials, experimental systems and methods used in many studies. Here, indicate whether each material, system or method listed is relevant to your study. If you are not sure if a list item applies to your research, read the appropriate section before selecting a response.

Materials & experimental systems

n/a	Involved in the study	
[ ]	[x]	Antibodies
[x]	[ ]	Eukaryotic cell lines
[x]	[ ]	Palaeontology
[ ]	[x]	Animals and other organisms
[x]	[ ]	Human research participants
[x]	[ ]	Clinical data

Methods

n/a	Involved in the study	
[x]	[ ]	ChIP-seq
[ ]	[x]	Flow cytometry
[x]	[ ]	MRI-based neuroimaging

Antibodies

Antibodies used

(Company: Biolegend ; Antigen-Fluorochrome: B220-PE-Cy7; Clone: RA3-6B2; Catalog Number: 103222)
(Company: Biolegend ; Antigen-Fluorochrome: B220-A700; Clone: RA3-6B2; Catalog Number: 103232)
(Company: BD; Antigen-Fluorochrome: CD138-BV711; Clone: 281-2; Catalog Number: 563193)
(Company: Biolegend ; Antigen-Fluorochrome: CD138-APC; Clone: 281-2; Catalog Number: 558626)
(Company: Biolegend ; Antigen-Fluorochrome: CD62L -BV605; Clone: MEL-14; Catalog Number: 104438)
(Company: Biolegend ; Antigen-Fluorochrome: CD62L -PerCPCy5.5; Clone: MEL-14; Catalog Number: 104432)
(Company: Biolegend ; Antigen-Fluorochrome: CD45.1-PE; Clone: A20; Catalog Number: 110708)
(Company: Biolegend ; Antigen-Fluorochrome: CD45.1-APC; Clone: A20; Catalog Number: 110714)
(Company: Tonbo Biosciences; Antigen-Fluorochrome: CD45.1-FITC; Clone: A20; Catalog Number: 35-0453-U500)
(Company: Tonbo Biosciences; Antigen-Fluorochrome: CD45.1-APC-Cy7; Clone: A20; Catalog Number: 25-0453-U100)
(Company: Biolegend ; Antigen-Fluorochrome: CD45.2-PE-Cy7; Clone: 104; Catalog Number: 109830)
(Company: Tonbo Biosciences; Antigen-Fluorochrome: CD45.2-PE; Clone: 104; Catalog Number: 50-0454-U100)
(Company: Biolegend ; Antigen-Fluorochrome: CD45.2-FITC; Clone: 104; Catalog Number: 109806)
(Company: Tonbo Biosciences; Antigen-Fluorochrome: CD45.2-PerCPCy5.5; Clone: 104; Catalog Number: 65-0454-U100)
(Company: Biolegend ; Antigen-Fluorochrome: CD45.2-APC; Clone: 104; Catalog Number: 109814)
(Company: Biolegend ; Antigen-Fluorochrome: CD11b-APC-Cy7; Clone: M1/70; Catalog Number: 101226)
(Company: Biolegend ; Antigen-Fluorochrome: F4/80-APC-Cy7; Clone: BM8; Catalog Number: 123118)

	(Company: Biolegend ; Antigen-Fluorochrome: CD90.2-APC-Cy7; Clone: 30-H12; Catalog Number: 105328)<br>(Company: ThermoFisher; Antigen-Fluorochrome: IRF4-PerCP-eFluor 710; Clone: 3E4; Catalog Number: 46-9858-82)<br>(Company: Southern Biotech; Antigen-Conjugate: Goat anti-mouse Ig-UNLB; Catalog Number: 1010-01)<br>(Company: Southern Biotech; Antigen-Conjugate: Purified mouse IgM-UNLB; Catalog Number: 5300-01B)<br>(Company: Southern Biotech; Antigen-Conjugate: Goat anti-mouse IgM-HRP; Catalog Number: 1021-05)<br>(Company: Southern Biotech; Antigen-Conjugate: Goat anti-mouse Ig-AP; Catalog Number: 1010-04)
Validation	All antibodies were purchased from commonly used companies, which performed the validation. All primary antibodies were confirmed by the manufacturer for use in flow cytometry and reactivity to mouse antigens. All validation statements, citations, antibody / antigen details are found on the companies websites.

Animals and other organisms

Policy information about studies involving animals; ARRIVE guidelines recommended for reporting animal research

Laboratory animals	Mice used in experiments were between 8-12 weeks of age, were age/gender matched, and all genders were equally represented throughout the experiments. Experimental mice were euthanized via carbon dioxide asphyxiation in accordance with AVMA Guidelines, 2020 edition. Animals were housed in specific pathogen-free caging with 12 hr light/dark cycles by the Emory Division of Animal Resources and all procedures were approved by the Emory Institutional Animal Care and Use Committee.
Wild animals	This study did not involve wild animals.
Field-collected samples	This study did not involve animals collected from the field.
Ethics oversight	All procedures were approved by the Emory Institutional Animal Care and Use Committee.

Note that full information on the approval of the study protocol must also be provided in the manuscript.

Flow Cytometry

Plots

Confirm that:

- [x] The axis labels state the marker and fluorochrome used (e.g. CD4-FITC).
- [x] The axis scales are clearly visible. Include numbers along axes only for bottom left plot of group (a 'group' is an analysis of identical markers).
- [x] All plots are contour plots with outliers or pseudocolor plots.
- [x] A numerical value for number of cells or percentage (with statistics) is provided.

Methodology

Sample preparation	Spleens were isolated from mice post-mortem, mechanically homogenized, and red blood cells lysed. Cells were stained at 1 million/100 microliters in FACS buffer (1X PBS, 2mM EDTA, and 1% BSA) and stained with Fc Block (BD; 553141) for 15 minutes, antibody-fluorophore conjugates for 30 minutes, and then washed with 10 volumes of FACs buffer. All stained cells used for flow cytometry analyses were fixed using 1% paraformaldehyde.
Instrument	Flow Cytometry: BDLSRFortessa X-20 (cat: 656385) or BD LSRII (cat: 339101), FACS: BD FACS Aria II
Software	Flow cytometry data were collected using BD FACSDiva v8.0 and analyzed using FlowJo v9.9.5 or v10.6.2
Cell population abundance	Purity checks on sorted cells were not performed due to cell number limitations, but the FACS ARIA was calibrated to 99.9% purity using fluorescent beads prior to each sort.
Gating strategy	Samples were pre-gated using the following strategy: (1) Lymphocytes based on SSC-A and FSC-A, (2) Single cells based on FSC-H and FSC-W, (3) Live cells based on Live-Dead staining using Zombie Yellow or Zombie NIR Fixable Viability Kit, (4) CD11b-F4/80-CD90.2- for B cells. For adoptive transfers, transferred populations were identified by CD45.1 or CD45.2 congenic markers.

- [x] Tick this box to confirm that a figure exemplifying the gating strategy is provided in the Supplementary Information.
