# Supplementary material for: Antibody-secreting cell destiny emerges during the initial stages of B-cell activation
Source: Nat Commun. 2020 Aug 10;11:3989. doi: 10.1038/s41467-020-17798-x (PMC7417592; doi:10.1038/s41467-020-17798-x)
Supplement: Supplementary file 3 — Description of Additional Supplementary Files [file 41467_2020_17798_MOESM3_ESM.pdf]

Description of Additional Supplementary Files

File Name: Supplementary Data 1

Description: Detailed list of all antibody/stains used in flow cytometry, ELISA, and ELISPOT experiments. List includes information about antibody clones, company, catalog numbers, dilutions, and specific notes when necessary for clarity.
